# Tradeoff Negotiation: The Importance of Getting in the Game: Comment on "Swiss-CHAT: Citizens Discuss Priorities for Swiss Health Insurance Coverage"

**DOI:** 10.15171/ijhpm.2018.86

**Published:** 2018-09-15

**Authors:** Ann Mongoven

**Affiliations:** Markkula Center for Applied Ethics, Santa Clara University, Santa Clara, CA, USA.

**Keywords:** Deliberation, Healthcare Rationing, Health Policy, Health Insurance, Tradeoff Negotiation

## Abstract

Swiss-CHAT’s playful approach to public rationing can be considered in terms of deliberative process design as well as in terms of health policy. The process’ forced negotiation of trade-offs exposed unexamined driving questions, and challenged prevalent presumptions about health care demand and about conditions of public reasoning that enable transparent rationing. While the experiment provided grounds for optimism that public deliberation can contribute to the design of fair insurance service-packages, it also left unanswered questions. What are the ethical and policy implications of non-consensuses? What is the presumed relationship between process and justice of outcome?

The boldly conceived and executed Swiss-CHAT experiment offers both reasons for optimism, and resources to support, public deliberation on tradeoffs among multiple public goods that cannot all be maximized—in health policy and beyond.^1^ While working toward its pragmatic goal, a community-forged consensus on an adequate basic insurance package, the process provocatively:

- challenged what may be the equivalent of “urban legends” in health policy;
- suggested avenues for further development of deliberative democracy in health policy;
- encouraged broader exploration of strategies to support explicit negotiation of tradeoffs among public goods.

Swiss-CHAT’s lessons urge pragmatic integration of diverse theoretical deliberative approaches in order to force participants’ engagement with tradeoffs. Several unanswered questions raised by the process became articulated through it.

- What is the presumed relationship between process and justice of outcome?
- How should the community address health-related values-conflicts resistant to negotiation through a deliberative process?
- What should be the procedural relationship between the exercise and policy-making?
- How adaptable is CHAT to diverse social and political environments?

## Lesson 1: Game (Beyond?) Theory

The mechanism that prompted presumption-challenging, policy-relevant conversations among the Swiss participants was elegantly simple: a visual game-board tool for tradeoff demarcation. The project literalized a metaphor from contemporary political theory calling for more “playful” politics.^2^

Of course, the design of the CHAT-board and of the competing scenarios representing tradeoffs among healthcare goods is not assumption-free. But one might call it both assumption-light and theory-light. The tradeoffs encapsulated have empirical bases as well as conceptual coherence from human life-course models. By asking people to begin by using stickers to indicate a first-stab budget-constrained basket of covered services, the process avoids initial political or philosophical biases in briefing materials. While the authors helpfully could have explained the directions provided to Swiss-Chat participants in more detail, it seems they were minimal. Participants were equally free to consider their and their loved ones’ actual experiences in health care; their biggest hopes and fears regarding health care; their philosophical, religious, or cultural conceptions of justice; or their general political commitments. The results suggest that an initial “free for all” in terms of allowed informing perspectives did not result in a “free for all” in the negative sense of a chaotic process, and ironically enabled rather than suppressed participants’ ability to grapple with the fact that healthcare is not free for all.

Blending liberal political theory’s emphasis on fair public process with communitarian and feminist inclusivity of narrative reasoning enhanced the deliberation. The biggest lesson: theory may be less important than getting people in the game.^2-4^

## Lesson 2: Support Tradeoff Negotiation by Making It Unavoidable

The CHAT-board enabled a visualization of tradeoffs that was supportive and fun. Its constructive use should invite additional experiments developing supportive tools for tradeoff negotiation. Unlike decision matrixes, the CHAT board does not treat goods to be traded-off as independent variables—and they clearly are not in the case of health services. However, decision matrixes enable a clear mathematical weighting of priorities that can be helpful for some kinds of deliberations.^5,6^ Deliberative polling techniques may use a variety of visual and feedback strategies to bring out previously inchoate tradeoffs over the course of polling rounds.^7^ Structured support for tradeoff negotiation stands as a great public need not only in health policy, but in civic education and political life broadly writ.

Of course, discerning tradeoffs is a crucial and effort-filled first step toward constructive tradeoff negotiation. The CHAT board did a lot of that work for participants, defensibly so assuming that tensions among different kinds of health goods generally have been well-gleaned by Swiss and international health policy analyses. For other kinds of emergent public issues, getting to a CHAT board might itself require a public deliberative process, with different kinds of creativity entailed.

The “rub” of comparing individual, small-group, and full-group sticker-maps and comments among rounds provided critical resources for participants. Wisely, the process facilitators assessed individual perspectives in both group and private settings. Regrettably, though, the authors said little about how they invited participants to move back and forth through the series of comparative progressions. How to address challenges entailed in those steps is not obvious, and has implications for wider public discourse.

## Lesson 3: What Can Happen When Forced to Negotiate?

### (a) The Biggest Underlying Questions Can Come to the Surface.

Explicit community consideration of tradeoffs embedded in public budgeting choices can create a dialectic between narrower pragmatic questions and the biggest questions of public goal. Clearly this occurred in Swiss-CHAT, which allowed the basic question to emerge: what is health insurance *for*, after all? The CHAT enabled a dramatic inversion of majority view on whether the primary goal of insurance is to cover routine health costs (initial majority view) or to address unpredictable serious illnesses and crises (post-exercise majority view). That inversion deserved more comment from the authors. What about the CHAT process precipitated it? Engaging with imagined alternate health futures, with current data and stories, or both? Did the change reflect a perceived re-balancing of goals of insurance, or a more radical challenge to the Swiss system? How did it “square” or not with the norming of current Swiss insurance policy that occurred over the course of the deliberation? On the face of it, this tables-turning reminds that public discourses ironically can develop historically with little attention to driving underlying questions.

### (b) Presumptions Can Be Exposed as Such

Certain equivalents of urban legends in health policy remain unidentified assumptions in many international discussions. Perhaps the most pervasive of these is the presumption that health care demand is infinite—the public will always want more health services without wanting to pay for them.^6,8,9^ If health needs are presumed unlimited, and everyone wants everything that could help themselves or their loved ones, health policy can be perceived to require a stance of public reason that suppresses personal choice for the sake of publicly justifiable rationing.Even burgeoning development of shared decision-making—which presents patient choice as itself the result of deliberation—has not eliminated presumptions that frame patient choice as an independent variable and assume people resist decreased “choice” as a means to cost-control or quality.^10^

Among the piquant results of the experiment, certainly one of the most striking was the willingness of participants to accept less choice for the sake of lower costs and greater health care value. Yet it is not clear whether they viewed this transformation as a simple fettering of choice or as a harnessing of choice to supportive processes. The authors do not describe how perceived connections between gatekeeping, practice guidelines, quality, and cost-effectiveness emerged through these brief game-driven deliberations. How to offer conceptual and empirical resources to a deliberative process without over-directing the process stands as a major challenge to process designers. This challenge is not erased by the gaming approach’s intentionally limited initial guidance of the facilitators, who clearly play thick subsequent roles. Indeed, it is clear that the designers conceived of educative and deliberative goals as intertwined. What kind of information about current insurance policy and metrics of Swiss healthcare cost, quality, and access were provided? How were they chosen? Was the process designed to allow participants to describe desired information? I hope the authors will address their response to the challenge of supporting-without-directing deliberation more fully in future writings.

Another myth-busting result was the willingness of participants to limit services for cost goals, and correspondingly to discuss explicitly what kinds of healthcare services they view as offering marginal benefit. Willingness to use the game to address tradeoffs between choice and other values, and between different kinds of healthcare services, resulted in attained consensus on a “sufficient” basic insurance package. The participants in Swiss-CHAT seemed less prone to magical thinking, and more capable of thinking “qua citizen” without instruction about how to do that, than urban legends presume.

Those accomplishments are particularly noteworthy since while the “urban legends” are often recited as reasons to promote public deliberation, they just as easily could be invoked to dismiss the potential of public deliberation—to argue that health policy needs insulated technocrats because public rationing is impossible to achieve. One virtue of the CHAT-board and its game-based seeding of deliberation may be the self-fulfilling tendency of its own presumption: people will be able to play, fairly.

While the myth-busting is notable, the “norming” of the actual Swiss basic insurance package through the process deserves more probing from the authors, who deliberately designed the initial CHAT-board to avoid any benediction of the *status quo*. The validation of current policy raises questions not only because some prevalent starting points differed significantly from the actual Swiss mandated basic insurance package, but also because the CHAT process revealed notable regional differences in priorities. Did final consensuses cluster around the *status quo* policy because the *status quo* adroitly represents a compromise balance among regions and individuals? Or does the “norming” effect raise red flags that the CHAT process may have been more inhibited than intended? (A post-question comparing perceived acceptability of participants’ hypothetical plan with perceived acceptability of the actual basic package would be a helpful probe).

## Question 1: What Is the Relationship Between Process and Justice of Outcome?

The facilitators never clarify whether they view the CHAT process as a form of pure procedural justice. In other words, they never state whether they accept the results of the process as fair by definition, or whether they presume a more complex relationship between justice of process and justice of outcome. That is a crucial point to address in order for the CHAT to be policy-informative, especially given that some values-conflicts remained resistant to negotiation.

## Question 2: What About Remaining Stark Conflicts?

Swiss-CHAT revealed some strong conflicts that remained un-negotiated. Table 6 is particularly illuminative: for several of the proposed cost-cutting strategies polled post-deliberation, nearly proportional percentages of participants rated a given strategy as alternately most or least acceptable—while no strategy was endorsed by a clear majority. Given the brevity of the Swiss-CHAT sessions, it is not clear whether the largest disagreements represent entrenched values differences, different referential experiences, or differential exposure to relevant facts. Swiss-CHAT does not answer questions about when and why deliberation should be considered complete, or what the implications of non-consensus are for just policy.

## Question 3: For Whom Is Participation?

Given the resource-intensiveness of Swiss-CHAT, it is important to ask how the experience of participants can be turned into a vicarious resource for the broader Swiss citizenry. How will the Swiss-CHAT experience be tapped in policy-making processes, broader public discourse including online democratic forums, or civic education?

Further research in the field fruitfully might address another question: does participation in a public-priority-setting exercise affect consideration of actual future personal healthcare decisions?

## Question 4: Can Swiss-CHAT Take SwissAir?

Swiss-CHAT adapted the previously-developed CHAT approach in new ways both by taking it to a new cultural venue, Switzerland, and by applying it to a specific policy goal—defining the basic insurance service-package that must be included across multiple insurance plans offered in Switzerland’s nationalized but dispersed health care financing system. While Switzerland may have more consensus on health policy goals than some countries, the Swiss-CHAT experience reminds that multi-lingualism and multi-culturalism underlie perceived common starting points in Swiss politics.

Can a theory-light game travel light to diverse political and organizational contexts? Could it be employed at pressure points for micro-distributional justice as well as macro (for example, within a specific insurance pool)? Could it be adapted to promote civic discussion in schools among countries that have different health care financing and delivery systems?

Ultimately, how “universal” are tensions among different kinds of goods in healthcare, amidst differential values-preferences? The CHAT’s simplicity is its beauty. Perhaps only time and use will tell if that simplicity proves desirably elementary. Perhaps a version of CHAT will become the health-policy equivalent of “Fishbanks” (MIT’s open-source fishing simulation game designed to dramatize the problem of the commons).^11^

At any rate, the Swiss-CHAT experience makes international bystanders want to play. And that’s a good start.

## Ethical issues

Not applicable.

## Competing interests

Author declares that she has no competing interests.

## Author’s contribution

AM is the single author of the paper.
